# Meta-Analysis on the Effects of Transcranial Direct Current Stimulation on Naming of Elderly with Primary Progressive Aphasia

**DOI:** 10.3390/ijerph17031095

**Published:** 2020-02-09

**Authors:** Haewon Byeon

**Affiliations:** Department of Speech Language Pathology, School of Public Health, Honam University, 417, Eodeung-daero, Gwangsan-gu, Gwangju 62399, Korea; bhwpuma@naver.com; Tel.: +82-10-7404-6969

**Keywords:** brain stimulation, dementia, meta-analysis, naming, primary progressive aphasia, qualitative evaluation

## Abstract

*Purpose*: This study aimed to conduct a qualitative evaluation by synthesizing previous studies on the effect of transcranial direct current stimulation (tDCS) on primary progressive aphasia (PPA)’s naming ability and prove the effects of tDCS mediation on PPA naming using meta-analysis. *Methods*: This study searched literature published from January 2000 to July 2019 using four academic databases (i.e., PubMed, Web of Science, MEDLINE, and Cochrane Library). The final seven publications were systematically evaluated and meta-analysis was conducted for two papers. The effect size was estimated by a standard mean difference (SMD) using Hedge’s g, and the significance of effect size was confirmed using the 95% confidence interval. *Results*: The results of seven previous studies’ quality assessments ranged from 15 to 26, which were rated above adequate. The results of the meta-analysis showed that the effect size was 0.82 (95% CI: 0.16–1.47), which was a significant ‘large effect’. *Conclusions*: This meta-analysis proved that tDCS intervention significantly improved the naming performance of PPA. Future studies must confirm the effects of tDCS on naming intervention by using meta-analysis including many RCT studies.

## 1. Introduction

Naming is widely used as a representative screening test for determining communication disorders around the world. Naming is divided into confrontation naming and generative naming [1]. The confrontation naming requires the complex coordination of visual stimuli, object recognition, linguistic system, vocabulary system, and phonological production system and it is affected by the function of the temporal lobe [1]. If the brain area associated with word recall is damaged, the confrontation naming function will be compromised [2,3].

On the other hand, generative naming is an evaluation that produces words in a specific category during a given time [4]. It is composed of semantic fluency, which expresses words associated with a presented category (e.g., animal) voluntarily, and phonemic fluency, which speaks given phonemes (e.g., words beginning with ‘k’) voluntarily [5]. Generation naming, unlike confrontation naming requiring the role of the temporal lobe, is affected by the frontal lobe’s executive function, which searches for information and yields words using presented clues [6]. In particular, confrontation naming drew attention as an indicator for detecting neurolingual disorders as soon as possible and measuring the recovery of them because naming is the communication problem that commonly remains until the last recovery stage for patients with fluent aphasia and those with nonfluent aphasia [7]. It has been used as the most representative test for determining the communication problems of patients with neurolingual disorders [7].

Recent studies have reported that naming is one of the most prominent language problems due to dementia [8,9]. Although the degeneration of naming abilities occurs at a different stage depending on dementia types [10], it is a common language deficit of most dementia patients and naming issues are observed from the incipient stage [11]. Particularly, primary progressive aphasia (PPA), a type of dementia, is a neurological dysphasia associated with temporal lobe atrophy and it is different from other dementia types (e.g., Alzheimer’s disease) in the aspect that a language defect occurs ahead of a cognitive ability defect [12]. PPA draws attention because of language disorders such as naming, advance gradually, unlike the aphasia, a neurogenic language disorder. In other words, PPA gradually loses naming abilities such as verbal fluency while maintaining other communication abilities such as articulation ability. Therefore, naming is an important indicator in identifying and intervening PPA in the early stage and many researchers have been interested in this topic due to this reason [13].

On the other hand, the safety of brain stimulation such as tDCS, which stimulates the brain using electricity, has been proved and it has been widely used in the clinical coalface [14]. tDCS is brain stimulation stimulating a large area, unlike repetitive transcranial magnetic stimulation (rTMS) stimulating a small area intensively [15]. It has many advantages: It is inexpensive compared to rTMS, is portable because it is light, and does not require a specific posture in the course of treatment [15].

Many studies have proved the effectiveness of tDCS since 2010, and meta-studies are actively conducted in recent years to establish the basis of tDCS [16,17,18]. In the early stages of development, tDCS was used mainly in the fields of exercise rehabilitation and mental health (e.g., schizophrenia and depression) [17]. However, the use of tDCS tended to increase in recent years as a tool for the linguistic mediation of patients with a neurological impairment such as aphasia and dementia [18]. However, since the research trends to date are mainly limited to the fields of exercise rehabilitation and mental health, more studies are needed to prove the effectiveness of tDCS on dementia.

Up to date, the effects of tDCS on cognition and linguistic abilities are still controversial [19] and, above all, no common implications have been drawn to improve the language ability of PPA. Therefore, it is needed to prove the therapeutic effect of PPA scientifically. This study aimed to conduct a qualitative evaluation by synthesizing previous studies on the effect of tDCS mediation on PPA’s naming ability and prove the effects of tDCS mediation on PPA naming using meta-analysis.

## 2. Methods

This study carried out systematic analysis and meta-analysis in the process of research question selection, systematic literature search and selection, quality evaluation of literature, data extraction and coding, data analysis, and result report preparation.

### 2.1. Literature Search

This study searched literature published from January 2000 to July 2019 using four academic databases (i.e., PubMed, Web of Science, MEDLINE, and Cochrane Library). The search terms included ‘Dementia’, ‘Primary progressive aphasia’, ‘Neurodegenerative diseases’, ‘Transcranial direct current stimulation’, ‘tDCS’, ‘Naming’, ‘Generative naming’, ‘Naming ability’, ‘Confrontational naming’, ‘Responsive naming’, ‘Semantic fluency’, ‘Verbal fluency’, ‘Phonemic fluency’, ‘Executive function’, ‘Cognitive rehabilitation’, ‘Cognitive training’, ‘Language recovery’, and ‘Language therapy’.

### 2.2. Literature Selection

The literature was selected based on the Patient–Intervention–Comparison–Outcome–Study design (PICOS) [20] of the PRISMA protocol. The selection and exclusion of the searched literature were conducted by three researchers independently. When there is a discrepancy in selection and exclusion, the three researchers discussed whether the publication should be included in or excluded from the systematic review or now. The inclusion criteria of this study were (1) studies conducted on PPA, (2) studies confirming the effects of tDCS, (3) experimental studies, and (4) studies published in English. This study excluded qualitative studies, unpublished publications including dissertations, and articles published in other languages such as French, German, and Chinese.

**Figure 1:**
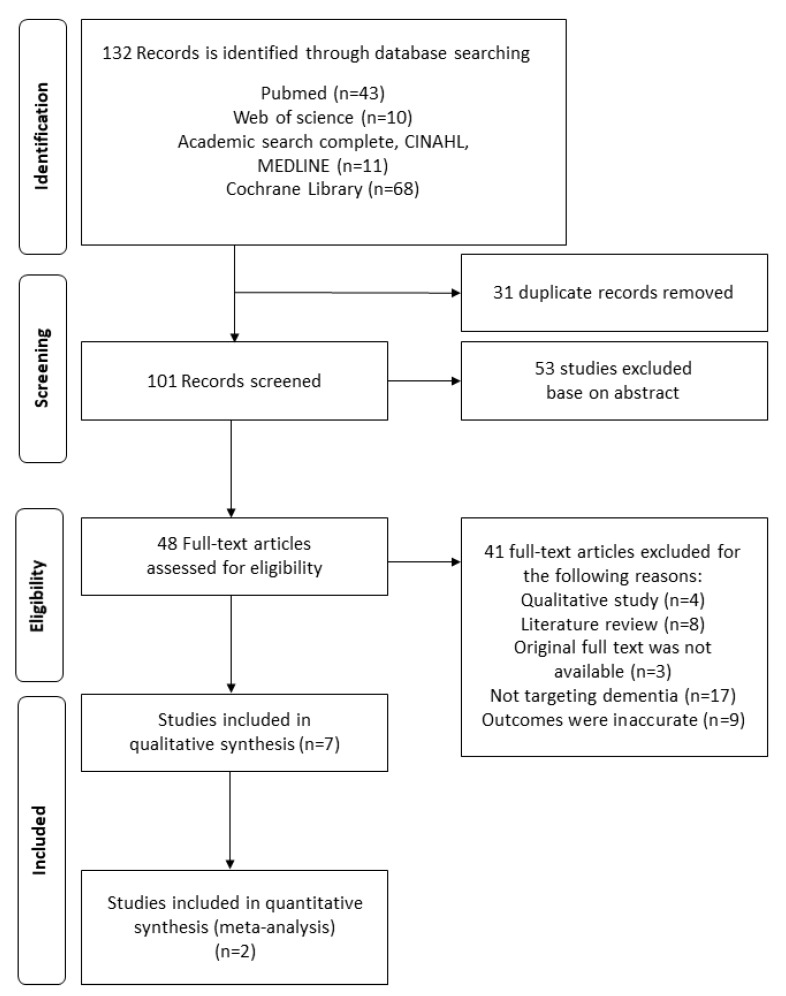
The flow diagram of this study.

This study found 132 publications in total. In the first step, 31 duplicated publications were excluded by comparing titles and abstracts. Moreover, 53 publications not related to the study topic were excluded. In the second step, the full texts of the remaining 48 publications were carefully examined and 41 publications were excluded. The excluded studies were non-experimental studies (n = 12), those without original full text (n = 3), those not evaluating dementia (n = 17), and those with inaccurate outcomes (n = 9). As a result, the final seven publications were systematically evaluated and meta-analysis was conducted for two papers, which we could extract representative values. The flow diagram of this study is shown in Figure 1.

### 2.3. Quality Assessment

This study used “Standard Quality Assessment Criteria for Evaluating Primary Research Papers from a Variety of Fields [21]” for quality assessment. This evaluation tool measured scores using a three-point scale (Yes = 2, Partial = 1, No = 0, N/A) and summed the scores of 14 evaluation items. The total score was converted into a percentage value and divided into strong (>80%), good (70–80%), adequate (50–69%), and limited (<50%) to examine the overall quality of studies [22]. The quality assessment of studies was performed by two researchers independently. If there is a discrepancy in the quality assessment item of a specific study, the final score was determined by discussion.

### 2.4. Meta-Analysis

This study extracted the analysis data of the selected publications and conducted meta-analyses for publications that could be statistically integrated using R version 3.4.2 (Foundation for Statistical Computing, Vienna, Austria). The representative values used for the analysis were estimated by calculating the difference between the treatment group’s mean and the control group’s mean and the mean differences normalized by standard deviations. The mean differences normalized by standard deviations were calculated according to Equation (1).

(1) $$\sqrt{S1_{pre}^{2}+S1_{post}^{2}-\left(2\times Corr\times S1_{pre}\times S1_{post}\right)}$$

The effect size was estimated by a standard mean difference (SMD) using Hedge’s g, and the significance of effect size was confirmed using the 95% confidence interval. The calculated effect size was interpreted as ‘small effect’ when it was smaller than 0.32, ‘middle effect’ when it was between 0.33 and 0.55, and ‘big effect’ when it was 0.56 or higher. Publication bias could not be estimated because target publications were less than 10.

## 3. Results

### 3.1. Quality Assessment Results

The quality assessment results of this study are presented in Table 1. The results of seven previous studies’ quality assessments ranged from 15 to 26, which were rated above adequate. All seven studies systematically presented the ‘objective of study’, ‘research design’, and ‘conclusion’ suitable for each item. Six studies, except one study [23], described the procedure of random allocation in the methodology section. While conducting studies, three studies [24,25,26] blinded researchers and four studies [24,26,27,28] blinded subjects. Six studies [23,24,25,26,27,28], except for [29], described the measurement methods and evaluation tools in detail. However, only one study [25] conducted a power test before starting the experiment. Additionally, only two studies controlled confounding variables [24,28].

### 3.2. Effects of tDCS on Improving the Naming Ability for PPA

The effects of tDCS on improving the naming ability for PPA were analyzed and the results are presented in Table 2. Ficek et al. (2018) [25] examined the combined effects of tDCS and speech therapy on 24 patients with PPA using letter accuracy. Their results showed that letter accuracy improved for the tDCS group and the placebo stimulation group but the improvement of the tDCS group was significantly larger. Hung et al. (2017) [23] evaluated the accuracy of naming by combining semantic feature training and tDCS intervention for patients with PPA and those with alzheimer’s disease (AD). Hung et al. (2017) [23] tested the intervention effect by dividing the results of the compounded intervention into trained items and untrained items. It was found that the trained items had higher accuracy than the untrained items after tDCS intervention and the effect was maintained until the follow-up period. Tsapkini et al. (2014) [27] evaluated the compound effects of spelling intervention and tDCS for six patients with PPA. In the untrained spelling item, the group which received tDCS and spelling intervention maintained the improved ability longer than the group which received placebo stimulation and spelling intervention. Tsapkini et al. (2018) also examined the combined effects of tDCS and naming/spelling intervention on 36 patients with PPA and reported that the trained items of the tDCS group were significantly improved immediately after the intervention. The difference between the trained words and the untrained words increased for the tDCS group and the placebo stimulation group as time goes on.

### 3.3. Meta-analysis for the Effects of tDCS Intervention on the Naming Performance of Patients with PPA

**Figure 2:**
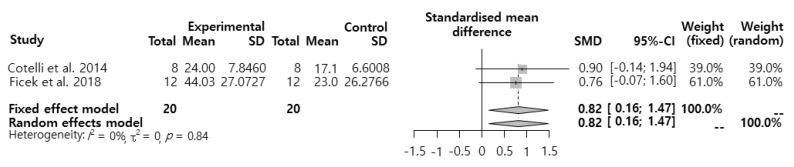
Effects of transcranial direct current stimulation (tDCS) intervention on patients with primary progressive aphasia (PPA’s) naming performance.

SMD about the effects of tDCS intervention on naming performance was analyzed (Figure 2). The results showed that the effect size was 0.82 (95% CI: 0.16–1.47), which was a significant ‘large effect’.

## 4. Discussion

This study conducted systematic reviews and meta-analysis to establish the scientific basis regarding the effect of tDCS on PPA’s naming ability based on literature published from January 2000 to May 2019. This study evaluated the quality of seven studies and found that, even though most of them were designed as RCT studies and blinded either researchers or subjects, only one study [25] conducted power analysis and only two studies controlled confounding variables [24,28]. Since the sample size bias has the possibility to distort the results of studies, it is recommended to carry out RCT studies that estimate sample size and control confounding variables before designing studies in the future.

This study conducted pre- and post-meta-analysis and found that tDCS intervention had a significant effect on improving PPA’s naming ability. PPA is a degenerative disease that causes linguistic problems such as naming ahead of cognitive problems such as orientation and visuospatial abilities [12,25,30]. The problem of naming ability is clearly observed from the incipient stage [12]. PPA may be classified as speech logopenic progressive aphasia, semantic dementia, or progressive nonfluent aphasia [31]. Naming ability decreases in patients with PPA regardless of PPA types [32]. PPA shows the deficiency of linguistic ability primarily and tDCS may have a significant effect on the PPA’s naming performance.

It is known that tDCS promotes and inhibits the spontaneous activity of the cranial nerve by stimulating with minute DC current through the scalp and making the DC reach the cerebral cortex [33]. In other words, tDCS stimulates the brain with a weak current below 2 mA to regulate the resting membrane potential voltage and induces changes in the spontaneous discharge rate of nerve cells and the activation of N-methyl-D-aspartic acid (NMDA) receptor [33]. However, how tDCS improves naming is not clearly known because the effectiveness of tDCS began to be evaluated in very recent years and there are no large-scale and long-term follow-up studies that evaluated the effects of tDCS on the improvement of naming [34]. Nevertheless, the results of this meta-analysis show that tDCS had a significant effect on improving PPA’s naming performance suggested tDCS could be an effective language mediator of PPA. Long-term follow-up studies will be needed to identify the effects of tDCS fully.

The importance of this study was that this study established the scientific foundation to evaluate the effects of tDCS on the naming ability of PPA. The limitations of this study are as follows. First, although this study collected and analyzed literature through various academic databases, this study only evaluated publications written in English and excluded papers written in other languages such as French and Chinese. Second, this study could not conduct a bias test because meta-analysis only analyzed two studies and there was a limit in proving the results. If the sample is small, the variance and standard deviation of individual studies become relatively large, which affects the confidence interval of the overall effect size and increases type II error. However, it is believed that the bias due to the small sample size was negligible because this study confirmed that the effects of tDCS intervention on PPA’s naming performance were a significant ‘big effect’. In the future, meta-analysis containing more samples is required.

## 5. Conclusions

This meta-analysis proved that tDCS intervention significantly improved the naming performance of PPA. However, the results should be generalized very carefully because the meta-analysis was conducted on only a few samples. Therefore, future studies must confirm the effects of tDCS on naming intervention by using meta-analysis using many RCT studies.

## Figures and Tables

**Table 1:** Results of the publication’s quality assessment.

	Criteria	1	2	3	4	5	6	7	8	9	10	11	12	13	14	Total
Study																
Wang, et al. 2013 [29]		+	+	±	+	N/A	N/A	N/A	+	−	−	+	±	±	+	15
Cotelli, et al. 2014b [24]		+	+	+	+	±	+	+	+	±	+	+	+	+	+	26
Tsapkini, et al. 2014 [27]		+	+	±	+	±	−	+	+	±	+	+	±	+	+	22
Hung, et al. 2017 [23]		+	+	±	+	−	N/A	N/A	+	±	+	±	±	+	+	18
McConathey, et al. 2017 [28]		+	+	+	+	±	−	+	+	±	+	+	+	+	+	24
Ficek, et al. 2018 [25]		+	+	±	+	±	+	+	+	+	+	+	±	+	+	25
Tsapkini, et al. 2018 [26]		+	+	±	±	±	+	+	+	±	±	+	±	+	+	22

+ = 2, ± = 1, − = 0.

**Table 2:** The effects of tDCS on naming improvement for PPA.

Study and Design	Participants	Intervention				Assessment	Outcomes
		Stimulated Region	tDCS	Sham tDCS	Session		
Ficek et al. (2018) [25] Blinding & Crossover & RCT Design	PPA (n = 24) tDCS (n = 12) : age = 65.2 ± 7.0 Sham (n = 12) : age = 69.1 ± 5.6	Left inferior frontal gyrus	Anodal 2 mA 20 min	30 s	15 sessions (daily)	Letter accuracy (Written naming)	Both tDCS and sham groups improved the letter accuracy of trained words
Hung et al. (2017) [23] pre-post design	PPA (n = 4) & AD (n = 1) : age = 66.6±8.56)	Left temporoparietal region	Anodal 1.5 mA 20 min	30 s	10 sessions (2 weeks)	Naming : six semantic items (trained and untrained items)	After tDCS intervention, trained items were maintain longer than untrained items.
Cotelli et al. (2014b) [24] Blinding & RCT design	PPA (n = 16) AtDCS (n = 8) : age = 63.4 ± 6.8 Placebo tDCS (n = 8) : age = 70.4 ± 6.8	Left dorsolateral prefrontal cortex	Anodal 2 mA 25 min	10 s	10 sessions (2 weeks)	Languistic abilities : Aachen Aphasia Tes (AAT)	Naming accuracy of the AtDCS group increased selectively during the pre–after intervention period.
McConathey et al. (2017) [28] Blinding & Crossover & RCT design	PPA (n = 15) : age = 68.71 ± 6.97 tDCS (n = 7, analysis n = 4), Sham (n = 8, analysis n = 3)	Left prefrontal region	Anodal 1.5 mA 20 min	30 s	10 sessions (2 weeks)	Sementic process : BNT, PPT, Category Fluency tests	Those with lower base scores have improved significantly since the actual tDCS compared to those with higher base scores.
Wang et al. (2013) [29] A1-B1-A2-B2	PPA (n = 1) : age = 67	Left posterior perisylvian region, left Broca’s area	B1–B2 Anodal 1.2 mA 20 min	A1–A2 30 s	5 days (A1–A2) 5 days (B1–B2)	Psycolinguistic Assessment in Chinese Aphasia (PACA)	After the B1 intervention, the scores of the four PACA sub items increased significantly.
Tsapkini et al. (2014) [27] Blinding & Crossover & RCT design	PPA(n = 6)	Left inferior frontal gyrus	1–2 mA 20 min	30 s	15 sessions	Number of correctly spelled word-prompts associated with each phoneme	Significant improvement has been maintained through the follow-up period under the tDCS.
Tsapkini et al. (2018) [26] Blinding & Crossover & RCT	PPA (n = 36) 1. tDCS (n = 20, crossover n = 15) 2. Sham (n = 16, crossover n = 15)	Left inferior frontal gyrus	2 mA 20 min	30 s	15 sessions (5 sessions per week)	Letter accuracy : trained & untrained items	Trained items were significantly improved immediately after tDCS intervention.
